# A de novo frameshift mutation in *ZEB2* causes polledness, abnormal skull shape, small body stature and subfertility in Fleckvieh cattle

**DOI:** 10.1038/s41598-020-73807-5

**Published:** 2020-10-12

**Authors:** Lilian J. Gehrke, Maulik Upadhyay, Kristin Heidrich, Elisabeth Kunz, Daniela Klaus-Halla, Frank Weber, Holm Zerbe, Doris Seichter, Alexander Graf, Stefan Krebs, Helmut Blum, Aurélien Capitan, Georg Thaller, Ivica Medugorac

**Affiliations:** 1 Institute of Animal Breeding and Husbandry, Christian-Albrechts-University of Kiel, 24098 Kiel, Germany; 2 IT Solutions for Animal Production (Vit), 27283 Verden, Germany; 3 Population Genomics Group, Department of Veterinary Sciences, Ludwig-Maximilians-University Munich, 80539 Munich, Germany; 4 Tierzuchtforschung E.V. München, 85586 Grub, Germany; 5 Clinic for Ruminants with Ambulatory and Herd Health Services, Centre for Clinical Veterinary Medicine, Ludwig-Maximilians-University Munich, 85764 Oberschleissheim, Germany; 6 Laboratory for Functional Genome Analysis, Gene Center, Ludwig-Maximilians-University Munich, 80539 Munich, Germany; 7 GABI, INRAE, AgroParisTech, Université Paris-Saclay, 78350 Jouy-en-Josas, France; 8 ALLICE, 750012 Paris, France

**Keywords:** Genetics, Animal breeding, Sequencing

## Abstract

Polledness in cattle is an autosomal dominant trait. Previous studies have revealed allelic heterogeneity at the *polled* locus and four different variants were identified, all in intergenic regions. In this study, we report a case of polled bull (FV-Polled1) born to horned parents, indicating a de novo origin of this polled condition. Using 50K genotyping and whole genome sequencing data, we identified on chromosome 2 an 11-bp deletion (AC_000159.1:g.52364063_52364073del; *Del11*) in the second exon of *ZEB2* gene as the causal mutation for this de novo polled condition. We predicted that the deletion would shorten the protein product of *ZEB2* by almost 91%. Moreover, we showed that all animals carrying *Del11* mutation displayed symptoms similar to Mowat-Wilson syndrome (MWS) in humans, which is also associated with genetic variations in *ZEB2*. The symptoms in cattle include delayed maturity, small body stature and abnormal shape of skull. This is the first report of a de novo dominant mutation affecting only *ZEB2* and associated with a genetic absence of horns. Therefore our results demonstrate undoubtedly that *ZEB2* plays an important role in the process of horn ontogenesis as well as in the regulation of overall development and growth of animals.

## Introduction

Genetic heterogeneity refers to a phenomenon where genetic variations at different loci (locus heterogeneity) or within the same locus (allelic heterogeneity) lead to a similar phenotype^1,2^. Furthermore, genes involved in heterogeneity may also affect other seemingly unrelated phenotypes, ultimately causing a pleiotropic effect^3^. While, in humans, researchers have reported heterogeneity for many clinical phenotypes^2,4,5^, such as breast cancer and hearing loss, in livestock, relatively few studies have reported genetic heterogeneity. For instance, Kijas et al.^6^ reported two different genetic variations in the gene *MC1R* that resulted in the dominant black coat color in three different pig breeds. In cattle, allelic heterogeneity is also observed for the polled condition (i.e. genetically hornless cattle)^7^ and double muscling (*MSTN*) in beef cattle^8,9^.

The development of horns in Bovidae, like cattle, goat, and sheep, is based on the interaction between different types of tissue starting with the differentiation of horn buds during embryogenesis and resumes with horn growth after birth^10^. The bony core of the horn originates from a separate ossification center in the dermis. As it grows, it progressively fuses with the frontal bone and becomes pneumatized by the action of osteoclasts coming from the frontal sinuses. The bony core is covered by a modified epithelium that produces the keratin sheath constituting the visible horn^10,11^. Previous studies have proposed that horn ontogenesis can serve as an adequate model to gain valuable insights on cell differentiation and intercommunication between bone and skin^12,13^.

Across the international cattle industry, dehorning of calves is commonplace. In Europe, more than 80% of dairy, 46% of beef and 67% of suckler calves are disbudded or dehorned for different practical and economic reasons, such as easier handling and a lower risk of injuries^14^. In the recent years, ethical issues about the routinely performed dehorning have been discussed in the scientific community as well as in the general society because dehorning is inevitably associated with stress and pain for the animals^15–18^. Therefore, polledness is a trait of great importance for the cattle industry and livestock science^19^ and it is constantly under scrutiny by the broad consumer community and animal welfare legislation^20^.

Multiple research groups mapped the *polled* locus on the proximal end of bovine chromosome 1 (BTA1)^21–23^. Finally, approximately 20 years after initial mapping^23^, it was discovered that the *polled* locus is characterized by allelic heterogeneity and four genetic variations causing polledness in cattle were identified^7,17,24–26^. None of these four structural variations affects a known coding or a regulating sequence. Additionally, Capitan et al.^12,27^ have also described two novel disorders involving atypical horn growth or polledness, and other symptoms. The first disorder, which they called Type 2 Scurs, is associated with a frameshift mutation in *TWIST1*, on BTA4. The second disorder, called Polled and Multisystemic Syndrome (PMS), is associated with a large deletion on BTA2 involving *ZEB2*, *ARHGAP15*, and *GTDC1* genes. Interestingly, the symptoms of PMS in cattle were similar to Mowat-Wilson syndrome (MWS) in humans, which is also associated with genetic variations in the human *ZEB2* gene. The MWS is characterized by distinctive facial features, postnatal growth retardation and variable congenital malformations including genitourinary anomalies. The most common genital anomaly in males is hypospadias followed by cryptorchidism^28^. In females, only one case of a vaginal septum is described^29^. Moreover, in humans, mutations in *TWIST1* causes Saethre-Chotzen syndrome, which is characterized by craniofacial and limb anomalies^30–32^. These findings of previous studies indicate that the development of horns is based on a complex network of interacting genes and that a better comprehension of this network could also provide valuable knowledge of genes that play a substantial role in the development of an organism.

In recent years, the advances and improvement in long-read sequencing technologies were quite useful in genomic applications such as in de novo assembly and identification of structural variations^33–35^. Moreover, these technologies can also provide a substantial gain in phasing the variants by generating information over long contiguous segments^36^. For instance, Stancu et al.^37^, using the MinION sequencer, identified a substantial proportion of SVs in two individuals that were not detected in short-read technologies. Moreover, by combining the variants identified using short-read information generated in the parents of one of the individuals, they also correctly assigned the long-reads of its parental origin.

In this study, we present the genetic and clinical characterization of a new genetic condition attributed to a de novo deletion in *ZEB2* gene that first arose in a Fleckvieh sire (FV-Polled1) and was later passed onto its offspring. This is the first report of a mutation affecting only *ZEB2* and associated with a genetic absence of horns. Additionally, in this study, we also utilized long reads sequencing technology to phase the causative mutation using the phased genotypes of 50K SNP array.

## Results

### A polled offspring born to horned parents showed abnormal phenotypes

**Figure 1:**
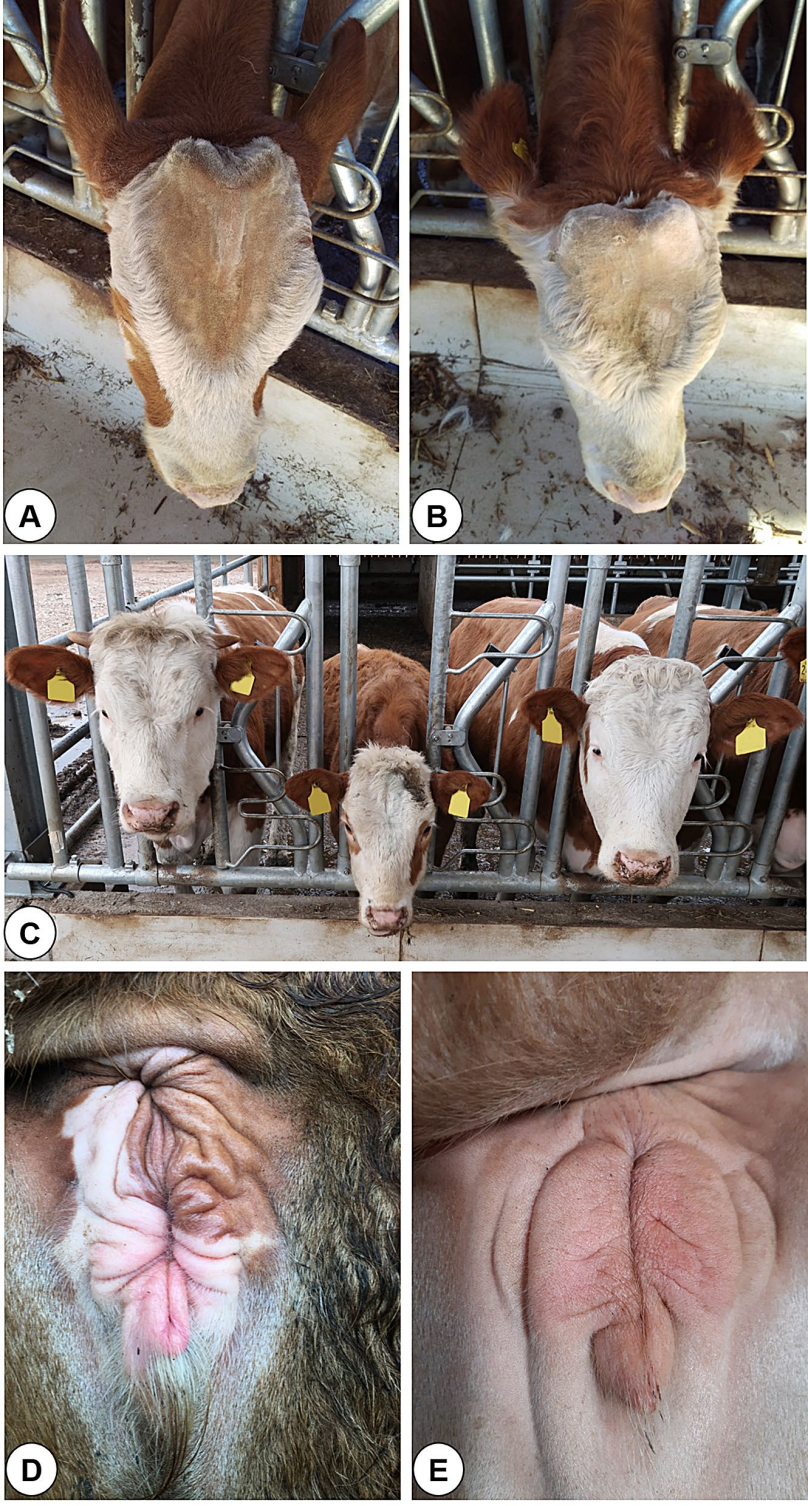
(**A)** Polled daughter of FV-Polled1 with the bony ridge along the frontal suture. (**B)** Wild-type dehorned daughter of FV-Polled1 without the bony ridge along the frontal suture. (**C)** FV-Polled1 daughter between two younger heifers kept on the same farm. The polled FV-Polled1 daughter (middle) was 456 days old at the time when the photo was taken. The horned FV-Polled1 daughter (left) was six days younger (450 days) and the dehorned daughter of another Fleckvieh bull (right) was 28 days younger (428 days). (**D)** Appearance of the vulva of a polled FV-Polled1 daughter; crimped labia with an overall “drawn-in” appearance. (**E)** Appearance of the vulva of a wild-type animal at the same age.

In this study, we present the case of a polled Fleckvieh sire (FV-Polled1) born to horned parents. It was hypothesized that the mutation responsible for the polledness in FV-Polled1 was de novo because polledness is an autosomal dominant phenotype, and parents and twin sister of FV-Polled1 were horned. The inheritance pattern of this mutation was studied based on 42 available FV-Polled1 offspring. The experienced staff of breeding advice service phenotyped FV-Polled1 and all its 42 offspring by close inspection and palpation of the skull and forehead. All the polled offspring, as well as FV-Polled1, were smoothly polled without any signs of epidermal keratinization or osseous bumps in the horn area. The results were verified by multiple inspections (refer to the “Methods” section for more details). For the sake of improved visibility, the horn bud and forehead of FV-Polled1 offspring was shaved and palpated. This enabled the discovery of an approximately 10 cm long and 1.5 cm prominent bony ridge along the frontal suture of polled animals. Importantly, this ridge was never observed in their horned half-sibs (see Fig. 1A,B).

**Figure 2:**
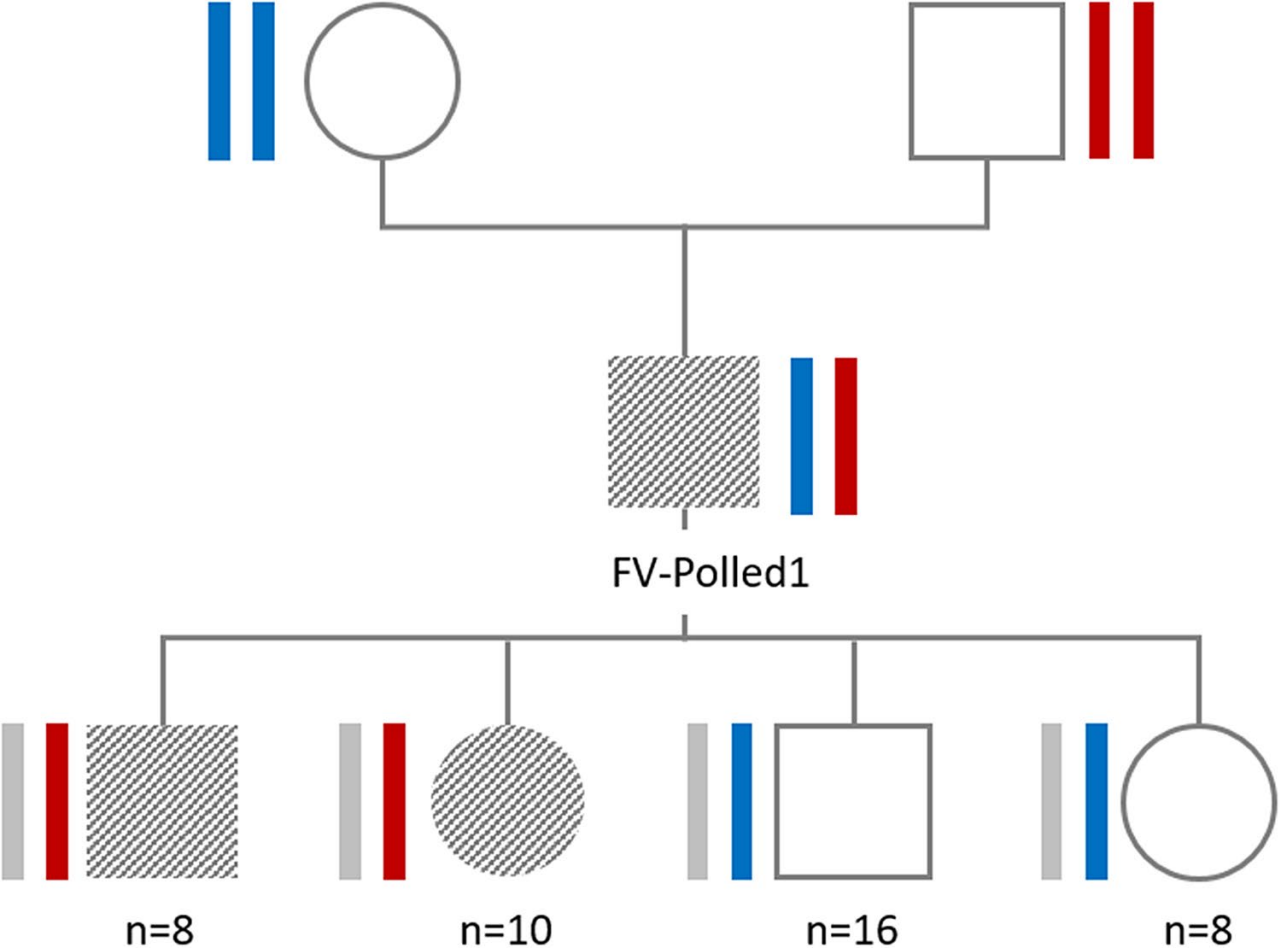
Pedigree information and haplotype structure of FV-Polled1, its parents and its progeny. Symbols to represent sex: a square represents male and a circle female individual; symbols and patterns to represent phenotypes: hatched grey shape represents polled and white shapes horned individuals; colors to represent haplotypes: red box represent paternal haplotypes and blue boxes the maternal haplotypes of FV-Polled1.

Of the 42 offspring of FV-Polled1, 24 were horned (male = 16, female = 8) and 18 were polled (male = 8, female = 10). These results suggested that the causal variant for the polled condition must be heterozygous in FV-Polled1. Further, Pearson’s chi-squared test^38^ showed that the ratio of polled and horned individuals did not deviate significantly from the expected ratio. Additionally, Fisher’s exact test^38^ confirmed that the horn phenotype was independent of sex, indicating monogenic autosomal dominant inheritance of the de novo mutation (see Fig. 2).

Additionally, we surveyed the FV-Polled1 offspring for any other apparent anomalies. A striking difference in body stature was noticed. Polled FV-Polled1 offspring were visually significantly smaller than horned offspring (see Fig. 1C). To confirm this, we measured the height at the withers, length from base of the neck to the base of the tail, length of labia majora and the bodyweight of 15 heifers descending from FV-Polled1 (eight polled and seven horned animals) at approximately equal age (see Table 1).

**Table 1:** Clinical ovarian findings, results of the progesterone level analysis and the final fertility status of polled and horned FV-Polled1 offspring at the end of the observation period.

Animal number	Horn status	Age at the time of the examination (in days)	Clinic (functional structures on the ovaries)	Progesterone	Final fertility status
1	Horned	606	Cl	+	Cyclic
2	Horned	550	n.ex	+	Pregnant
3	Horned	539	Cl	+	Cyclic/pregnant^a^
4	Horned	534	n.ex	+	Pregnant
5	Horned	480	Cl	+	Cyclic/pregnant^a^
6	Horned	445	None	+	Cyclic/pregnant^a^
7	Horned	437	None	+	Cyclic
8	Polled	598	n.ex	n.e	Pregnant
9	Polled	594	n.ex	+	Pregnant
10	Polled	559	None	−	Acyclic/pregnant^a^
11	Polled	444	None	−	Acyclic
12	Polled	443	None	−	Acyclic
13	Polled	422	None	−	Acyclic
14	Polled	421	None	+	Cyclic
15	Polled	419	None	−	Acyclic

^a^Positive pregnancy diagnosis at the end of the observation period.

*Cl*  corpus luteum, progesterone: +   at least one progesterone value > 1.0 ng/ml serum during the 4-week sampling period; −  no progesterone value > 1.0 ng/ml serum during the 4-week sampling period; *n.ex.*  not examined, *n.e.*  not evaluable.

The statistical analyses (Table S2) indicated that polled offspring of FV-Polled1 displayed significantly lower mean body weight (− 107 kg, *P* = 0.00014), lower mean height at the withers (− 20 cm, *P* = 0.00019) and shorter mean length from base of the neck to the base of the tail (− 8 cm, *P* = 0.00909) as compared with horned half-sibs. The mean length of the labia majora also differed between polled (LS-mean: 6.08 cm) and horned (LS-mean: 7.27 cm) animals (*P* = 0.0217). In addition, all polled animals had crimped labia, which gave the vulva an overall “drawn-in” appearance (see Fig. 1D,E). However, this appearance was also observed in one of the horned animals, which was not the offspring of FV-Polled1. Visual inspection and palpation of the teat system revealed one polled animal with a coarse, walnut-sized knot in the area of the right-sided rear udder quarter. The average vaginal length in polled animals (LS-mean: 29.2 cm) was observed to be smaller than in horned animals (LS-mean: 30.9 cm); this difference, however, was not statistically significant. A similar conclusion was drawn for average length of the cervix (LS-mean polled: 1.92 cm vs. LS-mean horned: 2.59 cm). However, significant differences (P < 0.05) between polled and horned animals were observed for the average diameter of the cervix (LS-mean polled: 0.92 vs. LS-mean horned: 1.62 cm) and the average diameter of the uterine horns (LS-mean polled: 1.29 vs. LS-mean horned: 1.75 cm). Results of the clinical examination of the ovaries and of the analysis of progesterone levels are shown in Table 1. Ovaries of animals that were pregnant at the time of the examination were not palpated.

At the end of the observation period, all seven horned animals were cyclic or pregnant. In the polled FV-Polled1 daughters, five animals were observed as acyclic, one as cyclic and two were pregnant. In the follow-up examination 8 months later, one of the acyclic animals was pregnant (No. 10). Three animals remained acyclic and one was dead (No. 13). In nutshell, all polled female offspring of FV-Polled1 showed a delayed onset of puberty.

### The de novo polledness locus maps to chromosome 2

**Figure 3:**
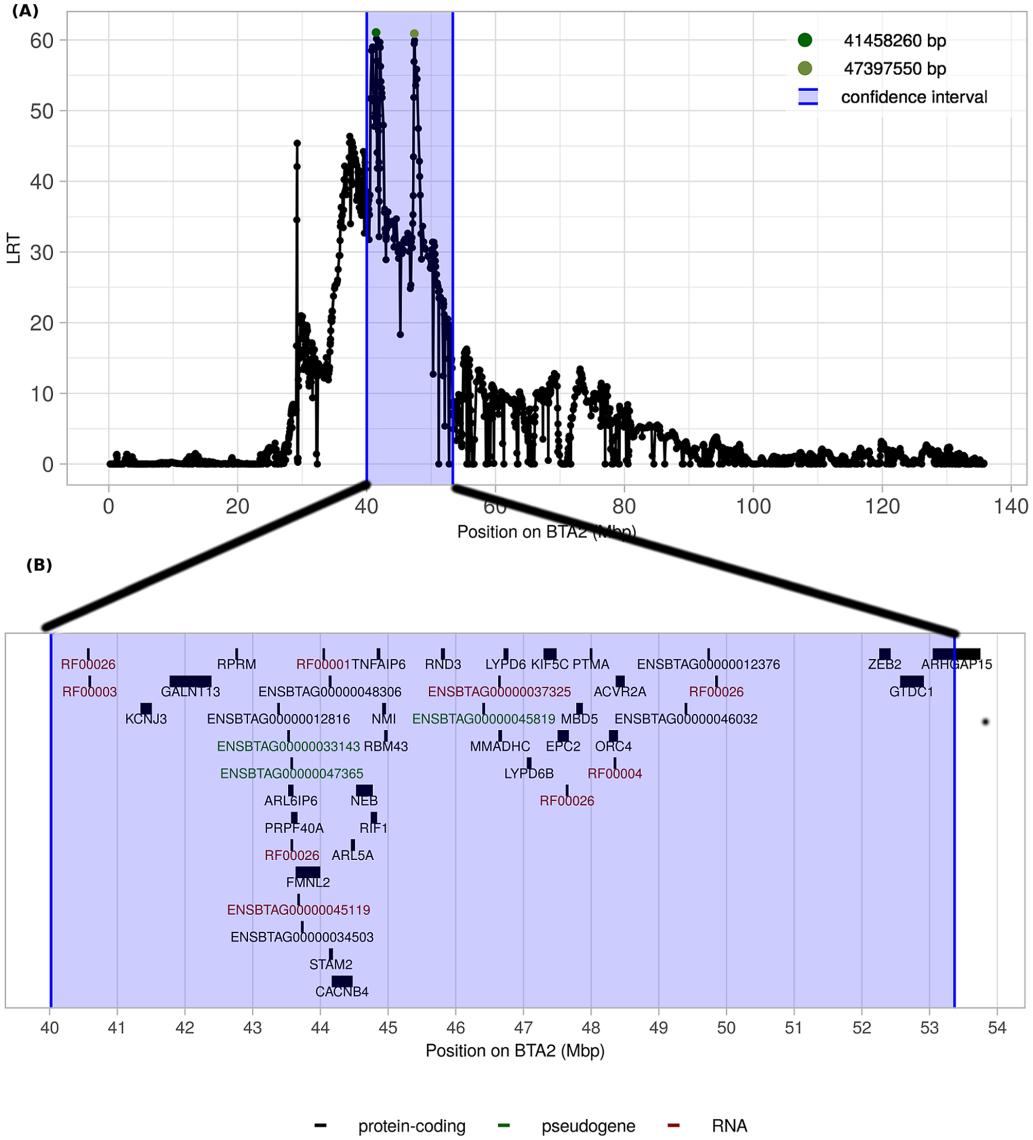
**(A)** Results of the combined linkage disequilibrium and linkage analysis of BTA2. Green dots indicate the window midpoint positions with the highest LRT values. The blue area indicates the confidence interval (CI) surrounding the two peaks. The CI is secured by one recombinant offspring on the distal and two recombinant offspring on the proximal end. **(B)** Detailed overview of the CI region including 32 protein-coding genes (black), 3 pseudogenes (green) and non-coding 9 RNA (red)^39,40^. (https://www.ensembl.org/index.html).

To map the genomic region associated with the de novo polled condition, 16 polled and 32 horned individuals were genotyped using a Bovine50K SNP array. After quality control, 39,489 SNPs were retained to map the genomic region by combined linkage disequilibrium and linkage analysis (cLDLA). The method identified only one strong signal that was traced to a haplotype located on BTA2 (Fig. S2). Likelihood-ratio test (LRT) values on BTA2 between 34.61 and 52.93 Mb exceeded the genome-wide significance threshold of *P* < 0.01 that indicated an association of this region with the observed polled condition. Two high peaks in close proximity were observed within the region, one with a maximum LRT value of 61.06 at 41,458,260 bp and the other at 47,397,550 bp with a maximum LRT value of 60.9 (Fig. 3A).

Phased SNP data of BTA2 of FV-Polled1, its parents and its offspring were inspected and the parental and maternal haplotypes deduced. The region was inspected for recombination events and reduced to a confidence interval (CI) from 40,021,512 to 53,369,279 bp, which is secured by one recombinant polled offspring on the distal and two recombinant polled offspring on the proximal end of the CI. For this region on BTA2, all polled offspring carried the common segment of the paternal haplotype of FV-Polled1, while all horned offspring carried the maternal FV-Polled1 haplotype (Fig. 2). Therefore, the de novo mutation must be located on the paternal haplotype of FV-Polled1. It must be taken into account that high quality of haplotypes is secured for all polled offspring by genotyping of trios, i.e. sire, dam and offspring. The functional annotation (Fig. 3B, Table S3) revealed that this ~ 13 Mbp region covered a total of 32 protein-coding genes, three pseudogenes, and nine non-coding RNAs.

### Identification of candidate de novo variants associated with polledness

To identify the de novo variant in the candidate region identified in the previous step, we sequenced the genome of FV-Polled1 with two different high throughput sequencing technologies: Illumina HiSeq paired-end sequencing and PromethION. Illumina paired-end sequencing generated about 397 million reads, while PromethION sequencing generated about 7.6 million reads with a mean read length of about 7 kbp. Both sequencing approaches generated sequenced data with an average depth of about 15 ×.

In the first step, variants were identified from Illumina sequences because PromethION long-read sequencing has a high error rate (~ 10%). In total, 27,453 variants were identified in the candidate region of ~ 13 Mbp on BTA2, using the algorithm implemented in *samtools mpileup*. Of these variants, 16,331 (15,233 SNPs and 1398 short insertions and deletions) were observed in a heterozygous state. In the subsequent filtering step, common variants between FV-Polled1 and individuals of 1000 Bull Genome Project were excluded. This comparison identified 30 indels and 8 SNPs that were solely present in FV-Polled1. Of these 38 candidate de novo variants (Table S4), 18 were intergenic, 16 were in introns of the various genes, one variant was upstream and two variants were downstream of genes, and one variant was in a coding region.

### A de novo 11-bp deletion in *ZEB2* is associated with polledness

**Figure 4:**
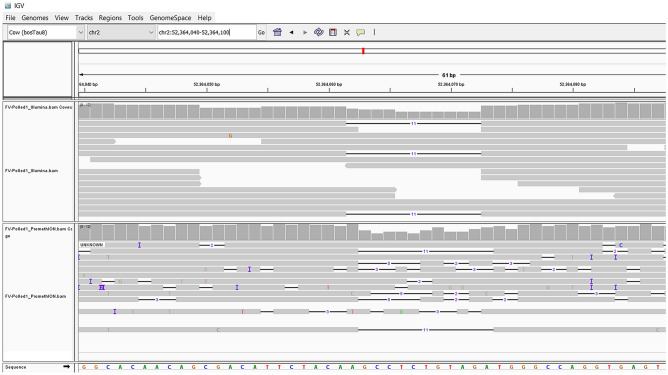
Screenshot of JBrowse showing 11-bp deletion identified in Illumina and PromethION sequences both.

Subsequently, each of these 38 candidate variants was sequenced using Sanger technology. Sequencing was carried out in FV-Polled1, its sire and two of its offspring (one polled and one horned). Interestingly, of the 38 candidate variants, only one was identified as true de novo mutation, i.e. present in FV-Polled1 and in the polled progeny but absent from the horned progeny and also from the sire of FV-Polled1 from which the BTA2 haplotype associated with polledness originates (Fig. 4). This de novo mutation was an 11-bp deletion, AC_000159.1:g.52364063_52364073del (*Del11*), located in the coding region of *ZEB2*. At the transcript level, this mutation can be written as NM_001076192.2:c.313_323del. To further show that the *Del11* has a de novo origin, we sequenced four SNPs located in the upstream and downstream sequences of this mutation. The results (Fig S1) indicated that, unlike *Del11*, all SNP alleles were shared between FV-Polled1 and its sire.

**Figure 5:**
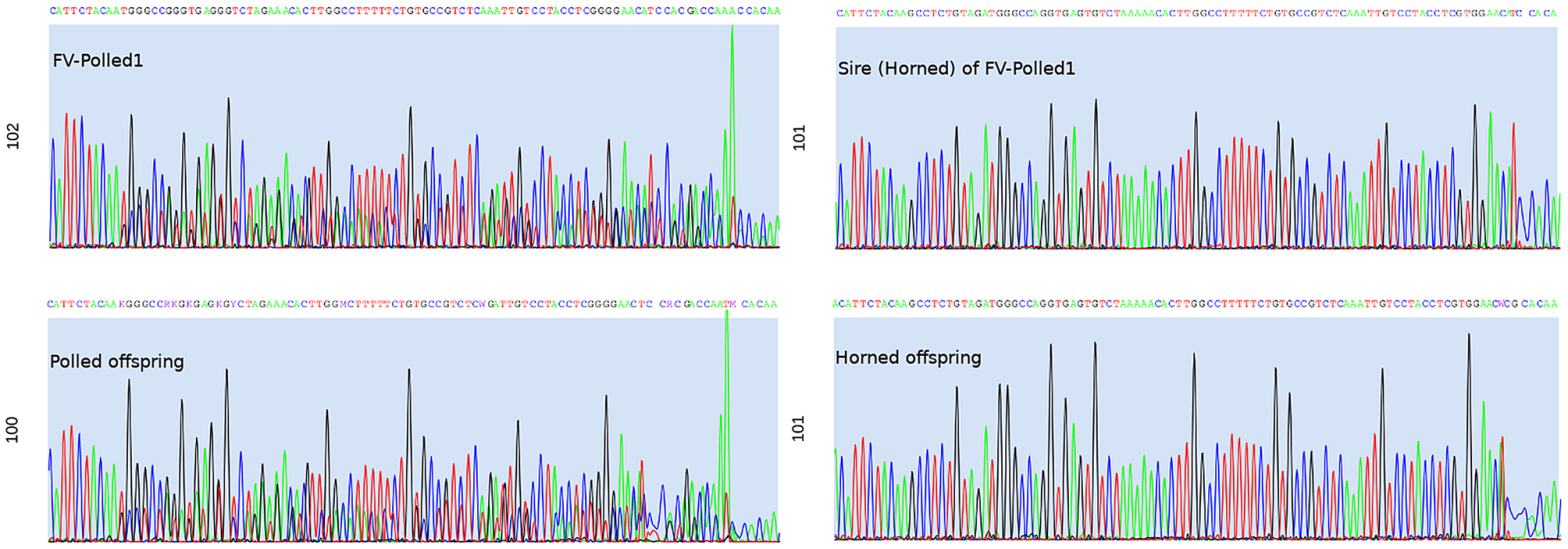
The chromatograms of the Sanger sequencing of the region involving *Del11*. Note that FV-Polled1 and its polled offspring are heterozygous for the deletion, while the horned offspring, as well as sire of FV-Poll, are homozygous for the wild allele.

Because *Del11* was identified by both the sequencing approaches, Illumina and PromethION (Fig. 5), we utilized the long reads sequenced by PromethION to phase the alleles.The phasing pipeline could only phase 13 of the 38 candidate variants. This low number was expected because the pipeline used phased SNPs from 50K SNP array as a “support” to phase the candidate variants and therefore, given the low density of markers in 50K SNP array, many promethION reads harboring candidate variants are not expected to contain phased SNPs. Nevertheless, a PromethION long read covering the heterozygous phased 50k position (position: 52,339,443), which was nearest to the genomic region at which *Del11*, was identified. Further analysis of this read suggested its maternal origin because it carried the maternal allele (“T” or “A”, depending on the strand) at the phased 50 k position. This maternal read carried the wild type allele at the site of deletion, confirming that *Del11* in FV-Polled1 has occurred in the paternal gametes.

### *Del11 *in *ZEB2* induces a frameshift mutation

**Figure 6:**
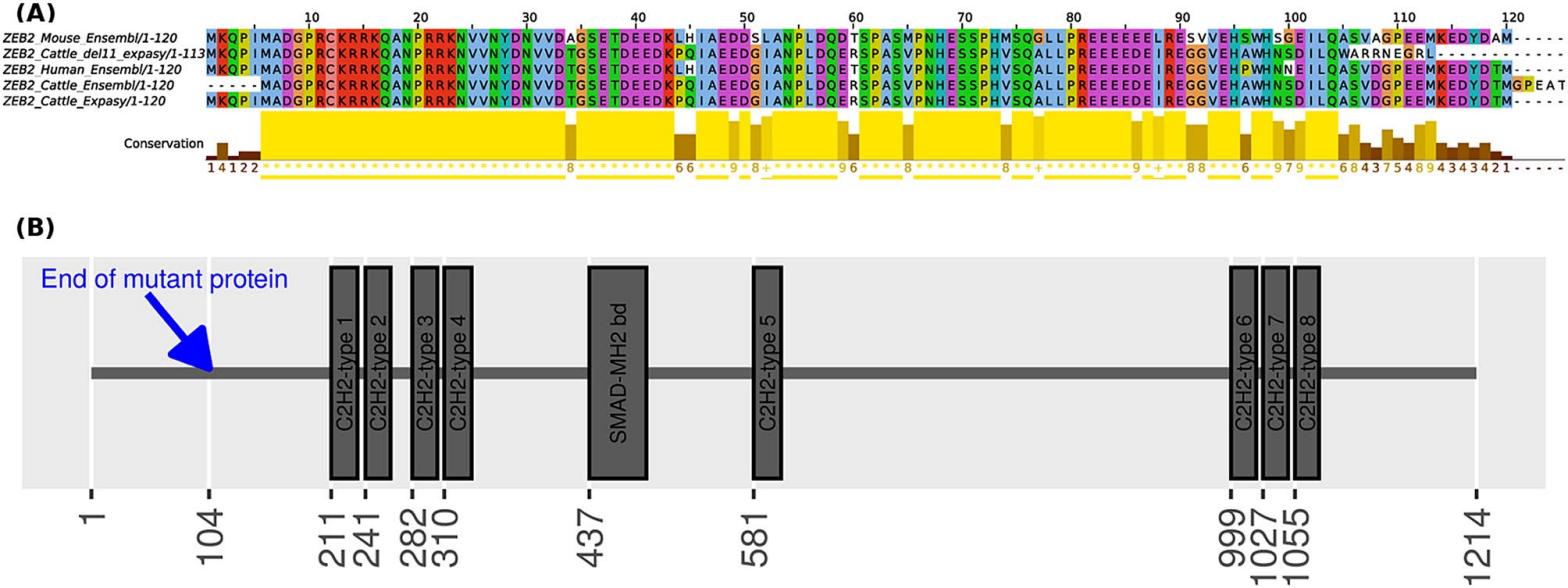
**(A)** ClustalW alignment of ZEB2 protein of human (ENSP00000487174), Mouse (ENSMUSP00000134849.1), and cattle (ENSBTAP00000053646.2). Additionally, ZEB2 protein predicted by ExPASy pipeline and the transcript containing *Del11* is also included in this multiple sequence alignment. Note that for the presentation purpose only the first 120 AA are shown. **(B)** Domain and region information of ZEB2 obtained from the UniProt database (https://www.uniprot.org/uniprot/Q9R0G7).

Bovine *ZEB2* (ENSBTAT00000061551.2) comprises 10 exons that encode a protein sequence (ENSBTAP00000053646.2) of 1209 amino acids. *Del11*, which was identified as de novo and compatible with the inheritance of the polled condition, is located in exon 2 (not counting 5′ UTR). Annotation by *VEP* predicted that *Del11* induces a translational frameshift (on the transcript predicted by Ensembl pipeline), which would alter the protein sequence from amino acid position 99 onwards. To better annotate *ZEB2* gene in UMD3.1 assembly and understand the precise consequences of the mutations, an independent annotation analysis was carried out. For this purpose, *ZEB2* (ENSBTAT00000061551.2) wild type cDNA was downloaded from Ensembl and translated into the expected protein using ExPASy. However, the transcription starting site (TSS), which was predicted by ExPASy, was located 15 bp upstream of the TSS predicted by Ensembl annotation pipeline. Moreover, a comparison of the protein sequence of *ZEB2* in cattle with its ortholog in humans (ENSP00000487174) indicated that annotation by ExPASy could be correct as both protein sequences start with identical amino acid sequences. Subsequently, the mutated cDNA, which contains *Del11*, was also translated into the expected protein using ExPASy and finally, both translated protein sequences were aligned using ClustalW2.1. The results (Fig. 6A) suggest that the deletion induces a frameshift which would lead to the premature termination of translation after the amino acid position 104 (as predicted by ExPASy tool). Consequently, the deletion results in a truncated protein (p.(Ala105Trpfs*10)) which is about 91% shorter than the wild type protein of *ZEB2*. Multiple alignments of the mouse (ENSMUSP00000134849.1), human and bovine ZEB2 proteins showed that coordinates in mouse and bovine orthologues are identical due to the strong conservation of the region (Fig. 6A). The mutant protein is predicted to lack all functional domains that are essentials for normal ZEB2 activity based on mouse annotations (see Fig. 6B).

## Discussion

Polledness in cattle is described as the complete absence of horns or any corneous growth in the horn area. Historical findings of the ancient paintings in Egypt suggest that polledness is a trait existing since several thousand years in the generally horned *Bovinae*^41^. Only recently, the *polled* locus was mapped to the proximal end of BTA1 spanning an interval of 325,239 bp. Furthermore, allelic heterogeneity was discovered at the locus, a phenomenon where different alleles of this locus cause an identical phenotype. So far, four different genetic variants have been identified^7,10,24–26^. The most common of these genetic variants-the ‘Celtic’ variant-is a 202 bp InDel event (1,705,834–1,706,045 bp). The ‘Friesian’ variant is a duplication-insertion event spanning approximately 80 kb (1,909,352–1,989,480 bp). The ‘Mongolian’ variant is a complex 219 bp duplication-insertion located within the region of the Friesian variant (starts at 1,976,128 bp). Just recently, the ‘Guarani’ genetic variation was proposed as a large duplication variant in Nellore cattle (1,893,790–2,004,553 bp). It is noteworthy that none of these genetic variants at the *polled* locus affects known coding or regulatory regions and therefore, the molecular mechanism leading to polledness remains unknown^7^.

Wiedemar et al.^17^ noted that de novo polled conditions could also arise in a population. Specifically, they described a case of a Limpurger bull, which was born to horned parents, transmitting the condition to its offspring. In the present study, we also report a case of polled individual (FV-Polled1), a Fleckvieh bull with horned parents. Moreover, FV-Polled1 transmitted the polled phenotype to its offspring, eliminating the possibility of a phenocopy or somatic mutation. Using multiple sequencing technologies the de novo mutation was identified as *Del11* (BTA2: 52,364,063–52,364,073), which was located inside the ~ 13 Mb region (between ~ 40 Mbp to  ~ 53 Mbp) identified as putative QTL by cLDLA using 50K SNP array data.

*Del11* encompassed the second exon of *ZEB2* and led to a translational frameshift (p.(Ala105Trpfs*10)). The frameshift caused premature termination of translation. Such a short transcript will either be degraded by nonsense-mediated RNA decay (NMD) or will generate a very short and nonfunctional peptide with length 91% shortened compared to the wild type ZEB2 protein. In fact, in humans, it is proposed that transcript from *ZEB2* with intragenic variants are expected mostly to undergo NDM or will generate a short nonfunctional peptides^42^. Based on the multiple alignment between orthologous ZEB2 proteins, it can be hypothesized that in case of a generation of the truncated bovine ZEB2 protein, it will lack all domains that are essential for its normal functioning. Genetic variations in *ZEB2* may cause multiple congenital anomalies. For instance, in humans, deleterious de novo heterozygous variants of *ZEB2* cause MWS. Distinctive facial appearances, moderate-to-severe intellectual disability, genital anomalies, and other multisystemic syndromes characterize the MWS. Most genetic variations in *ZEB2* causing MWS in human lead to haploinsufficiency through large deletions or premature stop codons^42^. Interestingly, Capitan et al.^12^ also reported a novel developmental disorder involving polledness—the Polled and Multisystemic syndrome (PMS)—in cattle which was associated with loss of heterozygosity of a very large (~ 3.7 Mb) genetic segment involving *ARHGAP15*, *GTDC1* and *ZEB2* genes. While they identified *ZEB2* gene as the most likely candidate in horn ontogenesis, they could not reject the association between the other two genes with horn ontogenesis. In the present study, we also observed a polled condition, which is due to the de novo deletion *Del11* identified in the second exon of *ZEB2*. Therefore, our results definitely confirm the role of *ZEB2* in horn ontogenesis. Moreover, we observed similar facial dimorphisms (polledness and bony ridge along the frontal suture) as described by Capitan et al.^12^. Further, Capitan et al.^12^ reported surprisingly low progesterone levels suggesting acyclicity and indications of premature ovarian failure. They proposed that these syndromes are also associated with a decreased expression of *ZEB2*. In the present study, clinical, biochemical and gynecological examinations revealed several distinctions between polled and horned daughters of FV-Polled1. Despite the similar age, all horned FV-Polled1 daughters were cyclic or pregnant, and about 120 kg heavier than the polled FV-Polled1 daughters. Moreover, gynecological examinations suggested various abnormal phenotypes related to fertility of polled daughter of FV-Polled1. However, except to crimped labia (Fig. 1D) none of these was systematic and thus less reliable.

In cattle, the main factor for the onset of puberty is bodyweight and not age. Therefore, delayed weight development results in delayed puberty, sexual maturity and breeding maturity. On average, Fleckvieh cattle reach sexual maturity at the age of seven months with a weight of 270 kg. First mating can be performed with a live weight of at least 400 kg, which corresponds to about 63% of the live weight of an adult cow and should be reached at the age of 16–18 months. This results in the age of 25–27 months at first calving for Fleckvieh cattle^43^. All examined polled animals were clearly underdeveloped and four of them not (yet) cyclic at the average age of 16 months, although they had already reached the necessary body weight for puberty. Additionally, all polled animals also had visible irregularities in the external genitals (crimped labia with an overall “drawn-in” impression). Despite the delayed development, three polled FV-Polled1 daughters became pregnant. One of those calved recently and had a low body weight, a very small udder and a low milk yield compared to fellow members of the herd.

The breeder himself designates FV-Polled1 bull and its polled offspring as quiet and well-behaved, however, it was not practical to implement any behavior test on his farm. The examinations concluded that polled offspring of FV-Polled1 displayed smaller body stature and lower body weight when compared to horned offspring. Interestingly, small body stature is one of the many symptoms frequently reported in patients of MWS in human and postnatal growth retardation was also described for PMS in cattle^10^. It is also interesting to note that mesenchymal-specific *ZEB2* knocked-out mice also displayed symptoms related to growth retardation^44^. These results indicated that *Del11* may have affected alternative spliced transcripts (isoform) of *ZEB2*, producing symptoms like polledness and short body stature but without affecting vital organs as reported in MWS in human or PMS in cattle^10^. We also note that the possibility of systematic sub-clinical symptoms that are too mild to observe cannot be ruled out in FV-Polled1 or its polled offspring.

The ontogenesis of horns is presumed to result from different tissues and their interaction suggesting a complex network of genes involved in the development of horns^45^. Previous studies have suggested that protein-coding genes, such as *FOXL2*, *RXFP2*, *TWIST1* and *ZEB2*, and non-coding RNA (LincRNA#1 and LincRNA#2), play a vital role in the differentiation of horn buds^10,17^. These protein-coding genes act as master regulators of epithelial- mesenchymal transition (EMT)—an important development process during which epithelial tissue acquire mesenchymal-tissue like properties. In fact, a possible role of EMT and neural crest cell migration in horn bud differentiation and horn growth has already been proposed by Capitan et al.^12^ and Wang et al.^46^. *ZEB2* encodes a Smad Interacting Protein 1 (SIP1), which is involved in processes like cell development and growth. It also plays a critical role in the development of connective tissues by forming the fine fibrils of collagen fibers^44^. Specifically, ZEB2 via SIP1 induces EMT by repressing the transcription of genes associated with the formation of a junctional protein that contributes to the dedifferentiated state of epithelia^47^. Further, it can be hypothesized that truncated ZEB2 protein lacked essential domains responsible for the differentiation of horn buds and consequentially, interrupted the EMT process leading to polledness in FV-Polled1 and its offspring^48^. Therefore, our results provide concrete evidence for the involvement of the *ZEB2* gene in horn ontogenesis.

In the present study, we used long-read data to determine the parental origin of a candidate de novo mutation. This was a crucial step as no DNA sample from the dam of FV-Polled1 was available for genotyping. The results indicated that the long read that was determined to be of maternal origin carried the wild type allele. Hence, this provides indirect but solid evidence that *Del11* must have originated in the germ cell of the paternal origin. It is worthwhile to note that the approach that we designed to determine the parental origin of the candidate allele is slightly different from Stancu et al.^37^. While Stancu et al.^37^ used phasing of heterozygous SNPs obtained from Illumina paired-end sequencing data of trio, we used phased genotypes of 50K SNP array data to determine the parental origin of candidate mutation. This was precisely the reason behind the observation that parental origin could only be determined for 13 of the 37 heterozygous candidate mutations.

To conclude, the results of this study identify *ZEB2* gene as a crucial factor in the complex genetic pathway involved in horn ontogenesis. Additionally, the present study demonstrates that combining results from multiple sequencing technologies is a powerful approach to identify and validate the genetic variants underlying Mendelian traits.

## Materials and methods

### Animals

FV-Polled1 was a polled Fleckvieh bull, which descended from horned parents. Its twin sister was horned as well. FV-Polled1 was mated by natural service to horned German Fleckvieh cows of a single farm to confirm the inheritance of the new polled phenotype. Experienced staff of breeding advice service examined the 42 available progenies of FV-Polled1 by visual inspection and palpation of the skull and forehead. All animals were inspected at last twice and most of these four times. As the farmer sells the majority of males at the age of eight weeks, the first two inspections were carried out at the age of two and eight weeks, respectively. Additionally, the third and fourth inspections were carried out at the age of approximately 1 year and 1.5 to 2 years, respectively. Note that third and fourth inspections concern all females, three males kept for farm’s own needs and FV-Polled1 itself. At the second or third inspection, the horn area of polled individuals was shaved to check for any small scurs or scabs.

Furthermore, we collected phenotypic data for 15 heifers of FV-Polled1 (eight polled, seven horned) at two different dates. All the selected heifers were older than 13 months and kept on the same farm. The height at the withers, length from base of the neck to the base of the tail and length of labia majora were measured by measuring stick while the bodyweight of each animal was estimated with a weight measuring tape. All 15 heifers were macroscopically examined for any other apparent anomaly. The differences between polled and horned animals with respect to the phenotypic traits described as above were assessed using the following linear models:

$${\text{y}}_{{\text{i}}} = \, \mu \, + {\text{ Age}}_{{\text{i}}} + {\text{HS}}_{{\text{i}}} + {\text{ e}}_{{\text{i}}}$$

where, *y*_*i*_ is the dependent variable related to body measurements of animal *i*, which is affected by fixed effects including the overall mean (*µ*), age at phenotyping (*Age*_*i*_ in days) and horn status (*HS*_*i*_, polled = 1 and horned = 2). The *e*_*i*_ represents random error of heifer *i*. The analysis was carried out using “aov” and “lm” function in R software.

### Clinical examination and blood sampling

A total of 15 female descendants of FV-Polled1 (eight polled and seven horned animals) were examined on the farm of origin. During visual inspection of the animals, the overall appearance was assessed with regard to male or female phenotypes. The gynecological examination included the visual inspection of the external genitalia, including length measurements of the labia majora and vagina, a vaginoscopy using a tubular speculum, the visual inspection and palpatory control of the teats as well as rectal palpation of the uterus and the ovaries, including transrectal ultrasonography (device KX5200, Co. Kaixin, 6.5 MHz linear probe). Additionally, blood samples were taken from the tail vein (Vena caudalis mediana) for analysis of the hormone level of progesterone. After refrigerated transport, the samples were centrifuged at 4000 × *g* for 10 min and refrigerated until progesterone analysis. Animals that could not clearly be assessed as cyclic at the time of the clinical examination were sampled three more times at intervals of 1 week.

Animals were categorized as “cyclic and/or pregnant” if they had functional ovaries or evidence of pregnancy during clinical examination, or if progesterone level exceeded the threshold of 1.0 ng/ml serum at least once during the 4-week sampling period. Animals that did not show any functional ovaries during the clinical examination and progesterone values lower than 1.0 ng/ml serum over the entire 4-week sampling period were categorized as “acyclic”.

### Genotypes

DNA was extracted from blood samples using the QIAamp DNA Blood Mini Kit from Qiagen and genotyped with the Illumina BovineSNP50 BeadChip (Illumina San Diego, USA). Physical SNP marker positions were determined according to UMD3.1 *Bos taurus* reference assembly^49^. For quality control, genotype data were filtered for the following criteria: (i) GenCall score higher than 0.95, (ii) missingness per marker less than 10%, (iii) missingness per individual less than 5%, (iv) minor allele frequency higher than 2.5%. After quality control, 39,489 SNPs remained for analysis.

### Mapping of the de novo polled locus

The initial mapping of the novel polled phenotype was performed on genotyping data of 48 animals. This mapping population was designed before all 42 progenies of FV-Polled1 were born and thus include only 27 of these. Table 2 describes the mapping population.

**Table 2:** Overview of the mapping population used for the initial mapping by combined linkage disequilibrium and linkage analysis.

	Polled		Horned	
	Male	Female	Male	Female
FV-Polled1 offspring	5	10	6	6
FV-Polled1	1	–		–
Sire of FV-Polled1	–	–	1	–
Twin sister of FV-Polled1	–	–	1	–
Maternal half-sister of FV-Polled1	–	–	–	1
Dams of FV-Polled1 offspring	–	–	–	13
Maternal grand-sires of FV-Polled1 offspring	–	–	4	–

Mapping of the de novo polled locus was carried out using combined linkage disequilibrium and linkage analysis (cLDLA) corresponding to the method proposed by Meuwissen et al.^50^. This procedure has already been described in Medugorac et al.^25^ and Gehrke et al.^51^. The procedure required the phase data; the inference of haplotypes and imputation of missing genotypes were carried out using hidden Markov models as implemented in BEAGLE 4.1^52^. In order to improve the accuracy of the analysis, genotype and pedigree information of 9548 additional animals genotyped with BovineSNP50K BeadChip, otherwise not included in this cLDLA, were added for haplotyping and imputation of mapping design. To account for the population structure in a mixed linear model (MLM), a unified additive relationship matrix (**G**) was calculated between all animals, and its inverse (**G**^-1^) was estimated^53^.

Subsequently, local haplotype relationships were calculated using a sliding window approach with overlapping windows and a window size of 40 sequential SNPs along each bovine chromosome. For each window midpoint, i.e. between marker 20 and 21, a local identity by descent (IBD) matrix was estimated as described by Meuwissen and Goddard^54^. Thereupon, the local IBD matrix was converted into a diplotype relationship matrix (**D**_**RM**_) as suggested by Lee and Van der Werf^55^.

cLDLA mapping of the de novo mutation was carried out with the horn status modeled as a quantitative trait with 1 for smoothly polled and 2 for horns. In this method, the linkage is accounted for in the reconstruction of haplotypes, while linkage disequilibrium is considered in the **D**_**RM**_^50^. Variance components analysis for each window midpoint was carried out with *ASReml*^56^ which estimates the maximum likelihood, variance components and fixed and random effects simultaneously by taking the IBD probabilities of the putative QTL into account. The following MLM was used:

$$\mathbf{y}=\mathbf{X}{\varvec{\upbeta}}+{\mathbf{Z}}_{1}\mathbf{u}+{\mathbf{Z}}_{2}\mathbf{q}+\mathbf{e}$$

where **y** is the vector of horn phenotypes converted into a quantitative trait, **β** is the vector of fixed effects including the overall mean (µ), **u** is a vector of n random polygenic effects for each animal with **u** ~ *N*(*0, Gσ*_*u*_^*2*^), **q** is a vector of random additive genetic effects of the putative QTL based on the **D**_**RM**_ with **q** ~ *N*(*0, D*_***RMp***_*σ*_*q*_^*2*^) where **D**_**RM**p_ is the **D**_**RM**_ matrix of the putative QTL at position *p*, i.e. midpoint between marker 20 and 21 of a sliding window along chromosomes. Random residual effects are included in the vector **e** with **e** ~ *N*(*0, Iσ*_***e***_^***2***^), where **I** is an identity matrix. Random effects (**u**, **q**, **e**) are assumed to be uncorrelated and normally distributed. Their respective variances (*σ*_u_^2^, *σ*_q_^2^, *σ*_e_^2^) were estimated simultaneously using *ASReml*. Fixed and random effects are related to by the incidence matrices **X,** **Z**_1,_ and **Z**_2_.

Finally, a likelihood ratio test (*LRT*) was calculated as a statistical test for the goodness-of-fit between the model without a QTL effect (null-hypothesis, *logL*(*H0*)) and the model including a QTL effect at position *p* (alternative hypothesis *logL*(*H1*)_*p*_), i.e. at each midpoint of sliding window along genome. The logarithms of likelihood estimated by *ASReml* were compared as follows:

$$LRT_{p} = \, - {2}*(logL(H0) \, - logL(H1)_{p} )$$

The *LRT* statistic is known to follow approximately a chi-squared distribution with one degree of freedom^57^. Since we used 987 (the number of informative SNP divided by window length, 39,489/40) independent windows in our analysis, the genome-wide *P*-value corrected for multiple testing was less than 0.0000101 (0.01/987) resulting in a significance threshold of LRT = 19.49.

After the candidate region was identified, the common haplotype present in FV-Polled1 and his polled offspring were identified. Visual inspection was carried out to determine the parental origin of the haplotype associated with de novo polled allele.

### Whole genome sequencing of FV-Polled1

Whole genome sequencing (WGS) of FV-Polled1 was carried out to identify the type of de novo variants present in the candidate regions associated with the de novo polled condition. For this purpose, Illumina paired-end read technology and PromethION long-read sequencing were used. To achieve an increased fraction of long DNA fragments, we extracted DNA immediately before long-read sequencing. For this purpose, the frozen blood sample collected from the individual FV-Polled1 was thawed and 100 µl were subjected to the QiaAmp Blood Mini Kit kit protocol (Qiagen, Hilden, Germany). Briefly, the resuspended blood was lysed and digested by proteinase K and RNAse A, bound to a column, washed and eluted by centrifugation.

### Illumina paired-end sequencing

The Library construction for the WGS was carried out with 200 ng of genomic DNA following the Illumina library preparation protocols (NexteraFlex DNA library kit, Illumina, San Diego, USA). The resulting amplified library was quantified and controlled on an Agilent Bioanalyser 2100 (Agilent, Santa Clara USA) and sequenced in 2*100 bp paired-end mode on an Illumina HiSeq1500, yielding approximately 200 Million read pairs.

BWA-mem was used to align the fastq files against the UMD3.1 reference genome. Finally, alignments of BTA2 were extracted from the bam file using the *samtools view*. Subsequently, the *samtools mpileup* was used with default settings to call indels and SNPs^58^ (command line : samtools mpileup -ugf Reference_genome.fa Sequenced_genome.bam | bcftools call -vmO z -o Sequenced_genome.vcf.gz).

### PromethION long-read sequencing

One µg of unsheared DNA was end-repaired and A-tailed with NEBnext UltraII End-repair and A-tailing module (New England Biolabs, Ipswich USA), purified with AmpureXP magnetic beads (Beckman Coulter, Brea USA), ligated to 1D sequencing adapters (LSK109 kit, Oxford Nanopore Technologies, Oxford, UK) and again purified with AmpureXP beads, according to the manufacturers’ protocols. In the last Ampure cleanup step, washing with 70% ethanol was replaced with washing buffer supplied by Oxford Nanopore Technologies (LSK109 kit). Approximately 400 ng of adapted DNA library was loaded to a primed PromethION flowcell and sequenced for 65 h on a PromethION Alpha sequencer.

Base calling of PromethION sequencing data was carried out using Albacore (v2.1.10) and Guppy v1.4.0 (ONT). Subsequent alignment against the UMD3.1 genome was performed with *ngmlr* using default parameters.

### Comparison against 1000 bull genome data

To identify the de novo variant responsible for the polled condition in the individual FV-Polled1 and its offspring, we used an approach similar to that described in Bourneuf et al.^59^. Briefly, we considered only the variants located in the mapping interval (BTA2: 40,021,512-53,369,279 bp) with a quality score and a mapping quality of more than 30. Then, assuming that the mutation occurred de novo and is found in the heterozygous state in the sequenced genome, we retained only heterozygous variants that were never observed in 2331 bovine control genomes from a broad variety of breeds, belonging to run6 of the 1000 bull genomes project^60,61^.

### Validation using Sanger sequencing

Genotyping was carried out by re-sequencing the PCR products obtained using a forward primer and a reverse primer designed to capture each candidate de novo variants (Table S1). The PCR products were directly sequenced using the PCR primers on an ABI 3170 capillary sequencer (Life Technologies). The sequenced data were analyzed using novosnp3.0.1^62^.

### Determining the parental origin of candidate mutations based on phased heterozygous SNPs of 50K array

As the inheritance pattern described earlier in the section suggested a paternal origin for the de novo polled mutation in FV-Polled1, we developed an in-house python script to determine the parental origin (PO) of candidate mutations. In brief, the python script identifies the mapped long-reads that cover the position of heterozygous phased SNPs and the position of candidate mutation; later, it uses this information to determine the PO of candidate mutations.

### Functional annotation

To annotate the gene content of the variants identified using *samtools*, Ensembl Variant Effect Predictor (VEP) was used^63^. Based on the genomic features overlapped by variants and the type of variants, the VEP predicts the associated consequences. Note that Ensemble release 94 (based on UMD3.1 assembly) was used for the functional annotation using VEP. Further, to estimate the effect of the candidate mutation, cDNA of bovine *ZEB2* was obtained from Ensembl Genome browser^39^. cDNA was kept in its wild type sequence and transformed in the mutated version, for which we manipulated the wild type cDNA sequence to contain the candidate mutation. Using ExPASy translate tool^64^, wild type and mutated cDNA were translated into its protein sequence. ClustalW 2.1 Multiple Sequence Alignment tool was used for the alignment (https://www.genome.jp/tools-bin/clustalw). Domain and region information on the ZEB2 orthologue in mouse was obtained from the UniProt database (https://www.uniprot.org/uniprot/Q9R0G7).

### Ethics statement

All work involving animals was conducted according to the national and international guidelines for animal welfare. Blood samples were collected with owner consent. Veterinarian examinations and sampling were approved by the ethics committee of Faculty of Veterinary Medicine-LMU Munich (179-26-06-2019) and ethics statement by the regional government of Upper Bavaria (55.2-1-54–2532.0-47-2016).

### Image source

Figure 1C was adapted. For aesthetical reasons color markings on the animal’s forehead were deleted and for privacy reasons the animal IDs were removed from the ear tags.

## Supplementary information

Supplementary Information.

## Data Availability

The BovineSNP50 BeadChip genotyping data of the Fleckvieh animals are submitted in Figshare with 10.6084/m9.figshare.13013885. The paired-end whole genome sequencing data of FV-Polled1 have been deposited in the European Nucleotide Archive (ENA) at EMBL-EBI under accession number PRJEB40548.
