# Supplementary material for: A de novo frameshift mutation in ZEB2 causes polledness, abnormal skull shape, small body stature and subfertility in Fleckvieh cattle
Source: Sci Rep. 2020 Oct 12;10:17032. doi: 10.1038/s41598-020-73807-5 (PMC7550345; doi:10.1038/s41598-020-73807-5)
Supplement: Supplementary file 1 — Supplementary Information. [file 41598_2020_73807_MOESM1_ESM.docx]

**A *de novo* frameshift mutation in *ZEB2* causes polledness, abnormal skull shape, small body stature and subfertility in Fleckvieh cattle**

Short title: A deletion in ZEB2 causes a polled phenotype in cattle

Lilian J. Gehrke^1,2¶^, Maulik Upadhyay^3¶^, Kristin Heidrich^3,4^, Elisabeth Kunz^3^, Daniela Klaus-Halla^5^, Frank Weber^5^, Holm Zerbe^5^, Doris Seichter^4^, Alexander Graf^6^, Stefan Krebs^6^, Helmut Blum^6^, Aurélien Capitan^7,8^, Georg Thaller^1^ and Ivica Medugorac^3, *^

^1^ Institute of Animal Breeding and Husbandry, Christian-Albrechts-University of Kiel, 24098 Kiel, Germany.

^2^ IT Solutions for Animal Production (vit), 27283 Verden, Germany.

^3^ Population Genomics Group, Department of Veterinary Sciences, Ludwig-Maximilians-University Munich, 80539 Munich, Germany.

^4^ Tierzuchtforschung e.V. München, 85586 Grub, Germany

^5^ Clinic for Ruminants with Ambulatory and Herd Health Services, Centre for Clinical Veterinary Medicine, Ludwig-Maximilians-University Munich, 85764, Oberschleissheim, Germany

^6^ Laboratory for Functional Genome Analysis, Gene Center, Ludwig-Maximilians-University Munich, 80539 Munich, Germany.

^7^ GABI, INRAE, AgroParisTech, Université Paris-Saclay, 78350 Jouy-en-Josas, France.

^8^ ALLICE, 750012 Paris, France

^¶^ contributed equally

^*^Corresponding author

## Supplementary Information


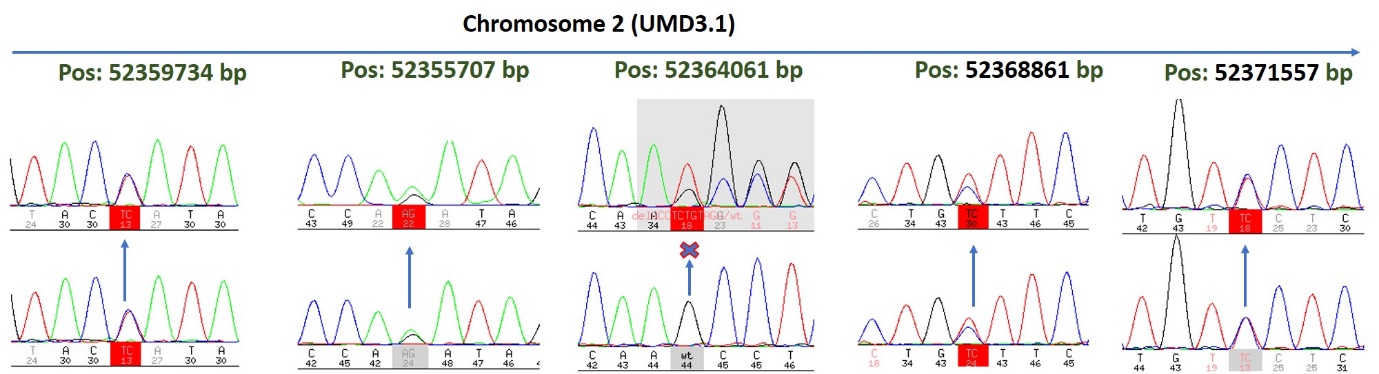


Fig S1. The figure shows that except the deletion identified at 52,364,061 on BTA2, all other variants are shared between FV-Polled1 and its sire, indicating *de novo* nature of this mutation.


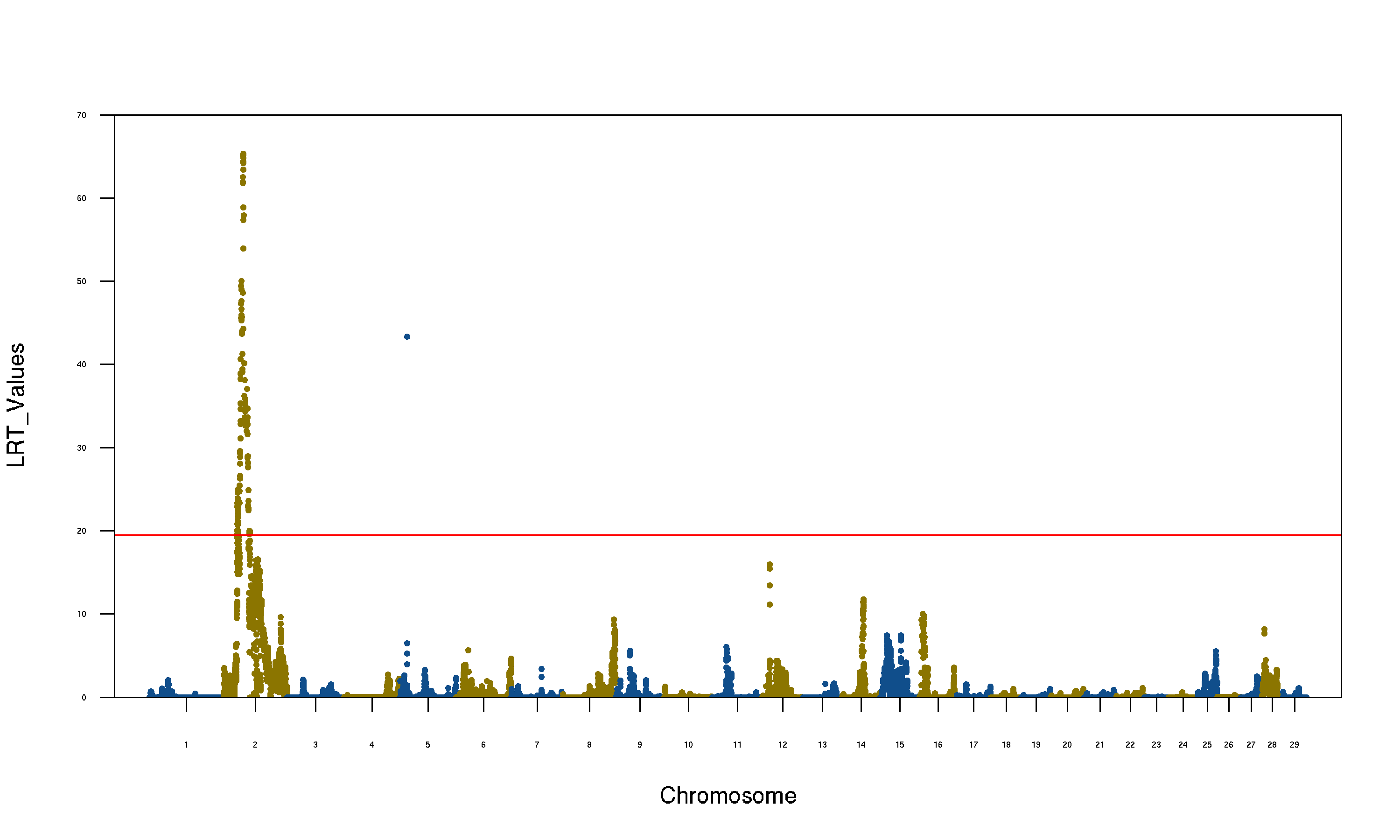


**Fig S2**. The results of the combined linkage disequilibrium and linkage analysis of entire genome. The red line displays the minimum threshold for significant LRT values.

Table S1. Primer sequences designed to capture and sequence each of the candidate *de-novo* variants

| Primer ID | Primer Sequences | Position on BTA2 | Reference | Alternative |
| --- | --- | --- | --- | --- |
| DS_Seq1_F | CAGCGTGCCTTTTTGTCAGT | 52368861 | T | C |
| DS_Seq1_R | GAATCCACATGACCCAGGAC |  |  |  |
| DS_Seq2_F | CCAGAGGAAGCAAATTGTAGTACC | 52371557 | T | C |
| DS-Seq2_R | CCTCATTTCCAAAGAATACACACA |  |  |  |
| US_Seq1_F | CGGCATCAGAAGACTTATTGG | 52359734 | T | C |
| US_Seq1_R | GACGGAAAATTTCAGCATTTG |  |  |  |
| US_Seq2_F | GCCTTATTTGAGCCACACCT | 52355707 | G | A |
| US_Seq2_R | TCATAGTTGCCCCACCCTAA |  |  |  |
| Novo01_F | TGAAGGAAGGAGGGTAAATGC | 40773434 | TGTG | TGTGGGTG |
| Novo01_R | TGAATTTCCCAATTTGTCAGG |  |  |  |
| Novo02_F | TGACCACCAAACCAAACAAA | 41106708 | AT | ATATT |
| Novo02_R | TTTCCCCTCCAGAGTGTTTTT |  |  |  |
| Novo03_F | TATTGGGGTTGGAGCAGGTA | 41508161 | GAAGTCAGGTATCAA | GAA |
| Novo03_R | TTCAAGAAACGCATCAGTGC |  |  |  |
| Novo04_F | AGGAGAGTGGATGCTGTTGG | 41544348 | CTG | CTGATG |
| Novo04_R | TGATGCATTGGAGAAGGAAA |  |  |  |
| Novo05_F | TGGACATAAAGATCATTGGAAAAA | 42170068 | TG | TGAG |
| Novo05_R | AAGAAATTGCTTCCCAGGTG |  |  |  |
| Novo06_F | TTTCCTTCTCCAATGCATGA | 43406755 | G | A |
| Novo06_R | AAAAATCCTGGCTTTACCTCC |  |  |  |
| Novo07_F | AGTTGGACAGGACCGAAGC | 43407268 | TATATTACAT | TAT |
| Novo07_R | TGCCTGGGTTCAAATATTCC |  |  |  |
| Novo09_F | CCTTGTTCGTGTTCAGTGTTTT | 43815085 | AA | AACACA |
| Novo09_R | CCTCGGTCACTGGTTTCTTAG |  |  |  |
| Novo08_F | AGGGCTCCTTGGAGAAATGT | 43780471 | AAAAAAA | AAAAAAAGAAAAAA |
| Novo08_R | CTGGGCAGGAAAGTGAACTC |  |  |  |
| Novo10_F | CACCACCATTTACACCACCA | 44238348 | C | CAA |
| Novo10_R | AGAGGGGAGTGAGCTTGTGA |  |  |  |
| Novo11_F | GGGAGGGAGACTGACTAGGG | 44238351 | ACGC | ACGCGC |
| Novo11_R | CGTGCTCAAAACAGGTGATG |  |  |  |
| Novo13_F | CTGTTCTGCTGGATGTGGAA | 44723863 | A | G |
| Novo13_R | CACCAGAAATAGAACCCTGCT |  |  |  |
| Novo14_F | CTGTGCGTTTTCACTGGAGA | 44960979 | TTA | TTATTCTA |
| Novo14_R | TGGGTTAAGTTCCTGGGTTG |  |  |  |
| Novo15_F | TTACAGCTTGCCCAGGATCT | 45057419 | G | GCAT |
| Novo15_R | CACCACAGCCTCTCACTCAA |  |  |  |
| Novo16_F | AGGTATGGAGGCGTTTGATG | 45276471 | A | G |
| Novo16_R | GGAGCGAGATTACTGGCTTG |  |  |  |
| Novo17_F | ACGCCTCTTGTTGATTCTCA | 46591893 | AAAAAAAAACAAAAA | AAAAAA |
| Novo17_R | TGAGGGAGGTGGTTTTTCAT |  |  |  |
| Novo18_F | ATCCTCCGACACCCTCTTTT | 46928989 | GCT | GCTACT |
| Novo18_R | TTACAGTCCCACCAGCAGTG |  |  |  |
| Novo06n_F | CCTAATGGGGTTCTTGCTGA | 43406755 | G | A |
| Novo06n_R | TAGGAACGGAGAAGGCAATG |  |  |  |
| Novo19_F | GGGGGAATAAGGGTAGTTGC | 47397223 | GCACTGCCTGTACCAGCATCTCACT | GCACT |
| Novo19_R | GGCAGAAGGCAAGTGTAGGT |  |  |  |
| Novo20_F | CCTTCTCCAGGGGATTTTTC | 48218691 | CAGA | CAGAAGA |
| Novo20_R | AGTTCAGGATGGGGAACACA |  |  |  |
| Novo21_F | TGGCATAGAAGTTTCAGCAAGA | 48671706 | AATTTTTT | A |
| Novo21_R | AGCAGATGAGCAGGAAGAGG |  |  |  |
| Novo22_F | GTGTCGCTTTTGTGTGCTGT | 48837238 | ACTGAACTGA | ACTGAACTGAGCTGAACTGA |
| Novo22_R | GCTGGGTCAGAAGCAGAGAG |  |  |  |
| Novo23_F | GCTGTATAATATCTCCTTTAGGTATGG | 49959182 | TATA | TATAGATA |
| Novo23_R | CCATCCATTACTTCTATTTCTAGTCCA |  |  |  |
| Novo24_F | GCACCCCACTCCAGTACTCT | 50554015 | CAGCAG | CAGCAGAAGCAG |
| Novo24_R | GGGTCTTTTCTCCATGTTTCTG |  |  |  |
| Novo25_F | AGGAAGAAACTCCCCATTCG | 50959341 | ATA | ATACTA |
| Novo25_R | AATCTCCCTTGGGTGGTTTT |  |  |  |
| Novo26_F | TGTGCCTGCAGTCATTTCA | 51944640 | ACA | ACATCA |
| Novo26_R | CTGGGGGAAAATCAATTCAC |  |  |  |
| Novo27_F | AATTGCTGTCCCATTTGCTG | 51980905 | TTTGCCAATTG | TTTG |
| Novo27_R | TCATTGTGATTGGCATCAAGA |  |  |  |
| Novo28_F | TCTGACTGAGGGGGAAACTG | 52054652 | T | C |
| Novo28_R | CCTTCTGGATGGTGTGGACT |  |  |  |
| Novo29_F | CCTTCTCCACAGCATTTCAA | 52054786 | T | C |
| Novo29_R | GTGTTTTTGAGGGGGATGAA |  |  |  |
| Novo30_F | TCTGACTGAGGGGGAAACTG | 52054792 | T | G |
| Novo30_R | GTGTTTTTGAGGGGGATGAA |  |  |  |
| Novo31_F | CCTGGGAAAAGGGCAATTAT | 52054806 | C | T |
| Novo31_R | GGCAGTAGTCTGTGGTCTTTTG |  |  |  |
| Novo32_F | TCTGACTGAGGGGGAAACTG | 52054808 | A | G |
| Novo32_R | GTGTTTTTGAGGGGGATGAA |  |  |  |
| Novo34_F | CCACTGTCCTGGAACATCCT | 52384619 | AA | AAACA |
| Novo34_R | GCTCTGACATGGCTTTCTCC |  |  |  |
| Novo36_F | AAAGGCGATCTACTCCAGCA | 52520238 | GCACA | GCACATCACA |
| Novo36_R | TAACCACCAAAACTGCCACA |  |  |  |
| Novo37_F | ATACTTTGGCCACCTGATGC | 52749062 | AACTGAA | AACTGAACTGGACTGAA |
| Novo37_R | GCAAAGGCATTGGATTTCAT |  |  |  |
| Novo38_F | GGGTTGCTAAGAGTCGGACA | 52847193 | AAAAAATAA | AAA |
| Novo38_R | GGATCTCAATTTGGCGTGTT |  |  |  |
| Novo20_2F | CTCCAGGGGATTTTTCCAAC | 48218691 | CAGA | CAGAAGA |
| Novo20_2R | ATGGCTCATAGTGTCAGAATGC |  |  |  |
| Novo29_2F | CCTGGGAAAAGGGCAATTAT | 52054786 | T | C |
| Novo29_2R | GGCAGTAGTCTGTGGTCTTTTG |  |  |  |
| Novo12_F | TCTTGAGCCATTTGATGTGC | 44496410 | GACATA | GA |
| Novo12_R | TTTCCTCATCTCTAGCAACC |  |  |  |
| Novo02_2F | TGAGGAAACGCCACAATAAA | 41106708 | AT | ATATT |
| Novo02_2R | TCCTATCTTCTGCCTCCGTTA |  |  |  |
| Novo06_3F | GCAACTTGCCCAATTTCCTA | 43406755 | G | A |
| Novo06_3R | CAGGGATCAAATCCACACCT |  |  |  |
| Novo14n_F | CTGGGTGGATTCCAGTGTCT | 44960979 | TTA | TTATTCTA |
| Novo14n_R | AAGAAAAGCAGGTGGCTGAC |  |  |  |
| Novo16_2F | TTCTCCACCTTGAGGGTCAC | 45276471 | A | G |
| Novo16_2R | TTCGTTTCCCCCTTTTATCC |  |  |  |
| Novo24_2F | TGCCATAATTCACCAAGGAA | 50554015 | CAGCAG | CAGCAGAAGCAG |
| Novo24_2R | TGGGTCTTTTCTCCATGTTTC |  |  |  |
| Novo04_2F | CCAAAATGAAACCTGCTTCC | 41544348 | CTG | CTGATG |
| Novo04_2R | ATGCCTGTGGTTGACAAGGT |  |  |  |
| Novo04_3F | GGGCAGGCAAAATATCAGAA | 41544348 | CTG | CTGATG |
| Novo04_3R | GCTGCTGACCAACGCTAAAT |  |  |  |
| Novo35_F | GTGTGTGGGGTCAGTGTTCA | 52508214 | CTG | CTGCTGATG |
| Novo35_R | GTCTCACAGAGTCGGACACG |  |  |  |
| Novo38_2F | TGTGGTTTTCACTTCCCACA | 52847193 | AAAAAATAA | AAA |
| Novo38_2R | GGATCTCAATTTGGCGTGTT |  |  |  |

**Table S2:** Least square means (LS-mean) with their confidence levels (Min, Max) and P-values of various phenotypic measurements taken from heifers of FV-Polled1 (n_polled_ = 8, n_horned_ = 7). All values are corrected for age at measurement that was significant (*P* < 0.01) for weight, length and height.

| Trait | Horn status | LS-mean | Confidence level | | P-value |  |
| --- | --- | --- | --- | --- | --- | --- |
|  |  |  | Min | Max |  |  |
| Weight (in kg) | Polled | 305 | 277 | 334 | 0.00014 |  |
|  | Horned | 412 | 382 | 443 |  |  |
| Length^*^ (in cm) | Polled | 110 | 106 | 114 | 0.00909 |  |
|  | Horned | 118 | 114 | 122 |  |  |
| Height^+^ (in cm) | Polled | 113 | 111 | 116 | 0.00019 |  |
|  | Horned | 133 | 119 | 125 |  |  |
| Length of labia majora (in cm) | Polled | 6.08 | 5.42 | 6.74 | 0.02170 |  |
|  | Horned | 7.27 | 6.56 | 7.98 |  |  |
| Length of vagina (in cm) | Polled | 29.2 | 26.9 | 31.4 | n.s. |  |
|  | Horned | 30.9 | 28.4 | 33.4 |  |  |
| Length of cervix (in cm) | Polled | 1.92 | 1.42 | 2.43 | n.s. |  |
|  | Horned | 2.59 | 2.04 | 3.15 |  |  |
| Diameter of cervix (in cm) | Polled | 0.92 | 0.68 | 1.28 | 0.01270 |  |
|  | Horned | 1.62 | 1.29 | 1.95 |  |  |
| Diameter of uterine horns (in cm) | Polled | 1.29 | 1.03 | 1.55 | 0.03070 |  |
|  | Horned | 1.75 | 1.46 | 2.03 |  |  |

*Length was measured from neck to the base of the tail of a heifer. ^+^Height was measured from wither of a heifer. n.s. non-significant.

Table S3. Genes, pseudogenes and RNAs located within the region of the confidence interval on BTA2. (Zerbino et al., 2018; https://www.ensembl.org/index.html)

| Name | Type | Start (bp) | End (bp) | Stable ID |
| --- | --- | --- | --- | --- |
| ACVR2A | protein-coding | 48381791 | 48476764 | ENSBTAG00000018114 |
| ARHGAP15 | protein-coding | 53065587 | 53732838 | ENSBTAG00000032289 |
| ARL5A | protein-coding | 44468905 | 44491772 | ENSBTAG00000002293 |
| ARL6IP6 | protein-coding | 43543016 | 43584232 | ENSBTAG00000001435 |
| CACNB4 | protein-coding | 44185984 | 44457832 | ENSBTAG00000002297 |
| ENSBTAG00000012376 | protein-coding | 49736040 | 49736399 | ENSBTAG00000012376 |
| ENSBTAG00000012816 | protein-coding | 43381652 | 43382632 | ENSBTAG00000012816 |
| ENSBTAG00000033143 | pseudogene | 43529729 | 43530624 | ENSBTAG00000033143 |
| ENSBTAG00000034503 | protein-coding | 43732220 | 43732799 | ENSBTAG00000034503 |
| ENSBTAG00000037325 | miRNA | 46645170 | 46645242 | ENSBTAG00000037325 |
| ENSBTAG00000045119 | miRNA | 43678327 | 43678408 | ENSBTAG00000045119 |
| ENSBTAG00000045819 | pseudogene | 46412021 | 46412626 | ENSBTAG00000045819 |
| ENSBTAG00000046032 | protein-coding | 49400488 | 49401260 | ENSBTAG00000046032 |
| ENSBTAG00000047365 | pseudogene | 43575860 | 43577277 | ENSBTAG00000047365 |
| ENSBTAG00000048306 | protein-coding | 44142619 | 44145088 | ENSBTAG00000048306 |
| EPC2 | protein-coding | 47524099 | 47651169 | ENSBTAG00000018581 |
| FMNL2 | protein-coding | 43652958 | 43979596 | ENSBTAG00000026851 |
| GALNT13 | protein-coding | 41791846 | 42371758 | ENSBTAG00000005562 |
| GTDC1 | protein-coding | 52584334 | 52897314 | ENSBTAG00000001132 |
| KCNJ3 | protein-coding | 41361137 | 41491255 | ENSBTAG00000006159 |
| KIF5C | protein-coding | 47315201 | 47472004 | ENSBTAG00000018125 |
| LYPD6 | protein-coding | 46723405 | 46759004 | ENSBTAG00000047426 |
| LYPD6B | protein-coding | 47068951 | 47099371 | ENSBTAG00000019683 |
| MBD5 | protein-coding | 47800355 | 47858327 | ENSBTAG00000019592 |
| MMADHC | protein-coding | 46648517 | 46664675 | ENSBTAG00000015962 |
| NEB | protein-coding | 44546002 | 44754701 | ENSBTAG00000006907 |
| NMI | protein-coding | 44930495 | 44950481 | ENSBTAG00000032369 |
| ORC4 | protein-coding | 48282736 | 48377766 | ENSBTAG00000015291 |
| PRPF40A | protein-coding | 43585376 | 43643096 | ENSBTAG00000048151 |
| PTMA | protein-coding | 47998614 | 47999416 | ENSBTAG00000016081 |
| RBM43 | protein-coding | 44961707 | 44973211 | ENSBTAG00000016217 |
| RF00001 | RNA | 44049602 | 44049693 | ENSBTAG00000044916 |
| RF00003 | snRNA | 40593052 | 40593207 | ENSBTAG00000028119 |
| RF00004 | snRNA | 48352291 | 48352468 | ENSBTAG00000036993 |
| RF00026 | snRNA | 40571290 | 40571396 | ENSBTAG00000043505 |
| RF00026 | snRNA | 43578069 | 43578172 | ENSBTAG00000042278 |
| RF00026 | snRNA | 47644965 | 47645071 | ENSBTAG00000042622 |
| RF00026 | snRNA | 49852590 | 49852696 | ENSBTAG00000044491 |
| RIF1 | protein-coding | 44763141 | 44818737 | ENSBTAG00000021020 |
| RND3 | protein-coding | 45800466 | 45822011 | ENSBTAG00000039731 |
| RPRM | protein-coding | 42762913 | 42764359 | ENSBTAG00000021526 |
| STAM2 | protein-coding | 44146496 | 44166620 | ENSBTAG00000001887 |
| TNFAIP6 | protein-coding | 44850892 | 44867293 | ENSBTAG00000007239 |
| ZEB2 | protein-coding | 52275074 | 52406207 | ENSBTAG00000012615 |

Table S4. 38 candidate *de-novo* variants identified on chromosome 2 in FV-Polled1.

| Position on BTA2 | Reference | Alternative |  |
| --- | --- | --- | --- |
| 40773434 | TGTG | TGTGGGTG | Intergenic |
| 41106708 | AT | ATATT | Intron |
| 41508161 | GAAGTCAGGTATCAA | GAA | Intergenic |
| 41544348 | CTG | CTGATG | Intergenic |
| 42170068 | TG | TGAG | Intron |
| 43406755 | G | A | Intron |
| 43407268 | TATATTACAT | TAT | Intron |
| 43780471 | AAAAAAA | AAAAAAAGAAAAAA | Intron |
| 43815085 | AA | AACACA | Intron |
| 44238348 | C | CAA | Intron |
| 44238351 | ACGC | ACGCGC | Intron |
| 44496410 | GACATA | GA | Downstream and 3‘UTR |
| 44723863 | A | G | Intron |
| 44960979 | TTA | TTATTCTA | Upstream |
| 45057419 | G | GCAT | Intergenic |
| 45276471 | A | G | Intergenic |
| 46591893 | AAAAAAAAACAAAAA | AAAAAA | Intron |
| 46928989 | GCT | GCTACT | Intergenic |
| 47397223 | GCACTGCCTGTACCAGCATCTCACT | GCACT | Intron |
| 48218691 | CAGA | CAGAAGA | Intron |
| 48671706 | AATTTTTT | A | Intron |
| 48837238 | ACTGAACTGA | ACTGAACTGAGCTGAACTGA | Intergenic |
| 49959182 | TATA | TATAGATA | Intergenic |
| 50554015 | CAGCAG | CAGCAGAAGCAG | Downstream |
| 50959341 | ATA | ATACTA | Intergenic |
| 51944640 | ACA | ACATCA | Intergenic |
| 51980905 | TTTGCCAATTG | TTTG | Intergenic |
| 52054652 | T | C | Intergenic |
| 52054786 | T | C | Intergenic |
| 52054792 | T | G | Intergenic |
| 52054806 | C | T | Intergenic |
| 52054808 | A | G | Intergenic |
| **52364061** | **AAGCCTCTGTAGA** | **AA** | **Frameshift** |
| 52384619 | AA | AAACA | Intron |
| 52508214 | CTG | CTGCTGATG | Intergenic |
| 52520238 | GCACA | GCACATCACA | Intergenic |
| 52749062 | AACTGAA | AACTGAACTGGACTGAA | Intron |
| 52847193 | AAAAAATAA | AAA | Intron |

11-bp deletion highlighted in grey.
